# Why are they not accessing it? User barriers to clinical information access

**DOI:** 10.5195/jmla.2021.1051

**Published:** 2021-01-01

**Authors:** Elizabeth Laera, Karen Gutzman, Angela Spencer, Charlotte Beyer, Saskia Bolore, John Gallagher, Sean Pidgeon, Ryan Rodriguez

**Affiliations:** 1 elizabeth.laera@bhsala.com, Medical Librarian, McMahon-Sibley Medical Library, Brookwood Baptist Health, Birmingham, AL; 2 karen.gutzman@northwestern.edu, Head, Research Assessment and Communications, Galter Health Sciences Library & Learning Center, Feinberg School of Medicine, Northwestern University, Chicago, IL; 3 angela.spencer@slu.edu, Assistant Professor, Medical Center Library, Saint Louis University, St. Louis MO; 4 charlotte.beyer@rosalindfranklin.edu, Library Director, Boxer Library, Rosalind Franklin University of Medicine and Science, North Chicago, IL; 5 saskia.bolore@ama-assn.org, Sales Manager, JAMA Network, American Medical Association, Chicago, IL; 6 john.gallagher@yale.edu, Director, Cushing/Whitney Medical Library, Yale University, New Haven, CT; 7 sean.pidgeon@oup.com, Publishing Director, Science & Medicine, Oxford University Press, New York, NY; 8 rrodriguez@bmj.com, Customer Engagement Manager, BMJ, Hoboken, NJ

## Abstract

The Medical Library Association's InSight Initiative provides an open and collaborative environment for library and industry partners to discuss vexing problems and find solutions to better serve their users. The initiative's fifth summit, continuing work from the previous summit, focused on understanding how users discover and access information in the clinical environment. During the summit, participants were divided into working groups and encouraged to create a tangible product as a result of their discussions. At the end of the summit, participants established a framework for understanding users' pain points, discussed possible solutions to those points, and received feedback on their work from an End User Advisory Board comprising physicians, clinical researchers, and clinical faculty in biomedicine. In addition to the pain point framework, participants are developing MLA InSight Initiative Learning content with modules to educate librarians and publishers about critical aspects of user behavior. The 2020 Insight Initiative Fall Forum will serve as a virtual home for constructive dialogue between health sciences librarians and publishers on improving discovery and access to information.

## INTRODUCTION

The Medical Library Association's (MLA's) InSight Initiative is a thought-leadership program, designed to bring library and industry partners together to work on the most vexing problems in these shared communities [1]. Through in-person and virtual summits, the Insight Initiative facilitates collaboration and builds trust while discussing big picture issues. MLA InSight Initiative Summit 3 focused on building bridges between health sciences information professionals and health information providers [2]. At the end of summit 3, participants determined that one of the most pressing problems that they currently faced was to better understand how users—such as physicians, clinical researchers, and biomedical clinical faculty—discover and access information.

Evidence-based information is essential to good patient care. From the earliest writings of Mesopotamia to today's apps that make information available at the point of care, reading, writing, and sharing knowledge about caring for the sick has always been of utmost importance. Studies have indicated that librarians and information resources are an integral part of this endeavor. In the Value Project funded by British Library Research and Development Department, 73% of clinicians received information from library resources that was immediately useful for their clinical decision making [3]. In one of the largest studies ever conducted on the value of libraries and information resources, 59% of physicians, residents, and nurses searching for information on patient care in electronic journals, PubMed/MEDLINE, and point-of-care tools, among many other resources, completely found what they were looking for, and over 95% of the more than 16,000 respondents found the information provided in the resources to be relevant, accurate, and current [4]. Furthermore, 33% said their choice of drugs was impacted by the information, and 19% said the information reduced unnecessary tests and procedures.

Accessing that information, however, is rife with complications. A 1985 study found that only 30% of physicians' information needs were met during a typical day [5]. In 2014, a systematic review analyzing 11 studies reported that clinicians generated about 1 question for every 2 patients and pursued answers to about 50% of them, but more than 20% of these questions went unanswered [6]. Frequently cited reasons for failing to answer clinical questions include lack of searching skills, accessibility to resources, or time as well as low motivation [7, 8].

## MEDICAL LIBRARY ASSOCIATION INSIGHT INITIATIVE SUMMITS 4 AND 5

InSight Initiative Summits 4 [9] and 5 [10, 11] divided participants into smaller groups to further define the issue of understanding how users discover and access information in the clinical environment. Participants collaborated on proposals to improve their joint understanding of user behavior and barriers. Each working group approached the problem in different ways and identified potential solutions, such as developing educational modules to address problems and setting up a forum dedicated to exchanging ideas and expressing views on user needs and behaviors. Two of the working groups joined and decided to focus on understanding and defining the barriers, or “pain points,” that users face when trying to access information [12]; they then brainstormed potential solutions for each point.

The working group defined pain points specifically as problems that limit a user's ability to access information. The group had the advantage of interacting with an End User Advisory Board (EUAB) composed of physicians, clinical researchers, and clinical faculty in biomedicine. These users, assembled as part of the InSight Initiative in response to summit 3 [2], shared their thoughts on access, discovery, and use of information resources. The end users provided feedback on the group's proposed pain points, helping to refine and identify the most pressing issues. The working group also brainstormed and discussed potential solutions for these pain points, based on their understanding of the literature, advice from the EUAB, and their expertise in industry publishing and library settings. This commentary outlines the main pain points (in no particular order), the potential solutions, and a call to action for library and industry partners.

## A FRAMEWORK FOR UNDERSTANDING USER PAIN POINTS

**Figure 1:**
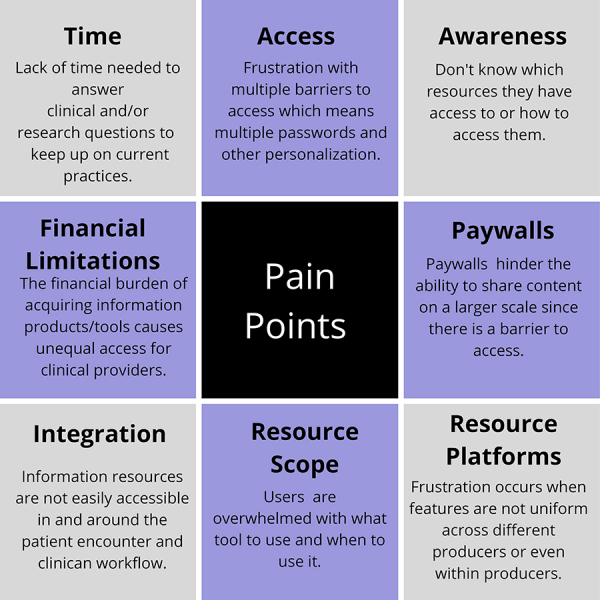
Descriptions of eight pain points that users experience when they try to access information in a clinical environment

The working group identified eight pain points that users experience when they try to access information in a clinical environment (Figure 1). These points were developed over the working group's series of conversations on topics such as supporting evidence-based medicine, user education for various platforms, institutional factors affecting access to resources, and authentication and privacy issues. Conversations with the EUAB refined the pain points and helped identify the most vexing problems. The advisory board later provided feedback on the final pain points while they discussed how potential solutions could affect workflow.

The first pain point identified is **time,** which includes time to get access, search, find, and receive help. Clinicians see many patients a day and spend a lot of time on documentation and reporting. Research has shown that clinicians lack the time needed to answer clinical questions and read up on useful and of-interest topics [7, 13]. Potential solutions include integrating information resources into the clinician workflow or electronic health record (EHR) [14], implementing library consult services [15], and providing continued yet improved, easy, and timely access to librarians and informationists. Institutions could provide scribe services, improving issues around the time spent on accessing information resources. “Everything would be impacted if the time pain point was alleviated,” according to one EUAB member. Others agreed that time was their biggest pain point when it came to accessing information.

The next pain point is **awareness** of the information resources that their institutions license and how these resources are accessed by users [16]. Librarians could improve awareness by expanding and improving upon current methods of advertising and instruction, which could be supported by publisher-created marketing materials that allow libraries to brand resources and materials [17, 18]. Collaboration between librarians and publishers on end-user marketing and training appeared to be key to alleviating this pain point. The EUAB members agreed that librarian participation in orientations and committees is an excellent way to remind clinicians of information resources.

**Access** is another pain point [7] and includes repeated login requests, too many passwords [19], and complex firewalls that block sites. The EUAB expressed frustration with multiple layers of authentication and personalization features that require an individualized login and password. Hospitals impose stricter Internet access, limiting users' ability to access information resources or search for needed information. Potential solutions include publishers buying in to federated authentication, such as RA21 [20], and institutions adopting a streamlined single sign on (SSO) mechanism. Librarians should develop good relationships with hospital and institutional information technology departments to limit blocked sites or multiple login demands when end users browse the Internet. Password managers utilized by end users could be a simple solution to a small aspect of this issue. While the access pain point has attracted a great deal of interest in the literature and from those working toward solutions [21, 22], the EUAB saw this as a low priority.

“**Paywalls** stop all investigations,” said one clinician. Another shared that nurses in their organization often resorted to finding illegal copies of paywalled literature [23]. The oft-complained-about paywall pain point encompasses the process of users hitting a paywall that limits access to full text literature [24, 25]. Even with effective SSO, users will still encounter paywalled content. Paywalls also hinder the ability to share content on a larger scale, a concept that the EUAB reiterated when they discussed sharing important clinical information, especially with colleagues at other institutions. Utilizing a tool such as Unpaywall or the Open Access Button could help busy clinicians. Improving verification of library resources via Internet protocol (IP) authentication beyond PubMed and Google Scholar, as well as educating end users on the many ways to obtain full text, are potential solutions that librarians can employ. Publishers could add an “Easy Button” or “Get It” button to get full text via interlibrary loan.

Issues involving too many platforms and lack of standardization across those platforms encompass the next pain point: **resource platforms.** Each publisher platform can have different search functions, rules of access, and accessibility [26]. Users may not search the full breadth of content that they have access to because they have to search across multiple platforms. Frustration occurs when features are not uniform across different producers or even within producers. Potential solutions include publishers working to ensure that their content is maximized for discoverability, especially by Google and Google Scholar, and make text and data mining a feature of their platforms. Standardization among publishers to build similar user experiences could also be helpful, although there was some discussion among both the EUAB and the working group about whether this was even possible. Additional solutions involve institutions and librarians implementing data and text mining initiatives that allow users to search for information across multiple platforms in the way that is most useful to them [27].

While the EUAB agreed that many different resource platforms are a pain point, the real pain point is understanding which tool to use and when, or **resource scope.** Users are overwhelmed with the amount of resources that are provided [28, 29]. Many resources are similar, and users do not have time to discern if a resource meets their needs. The simplicity and relatively limited features of Google make it successful, and the literature shows that clinicians often bypass traditional information sources in favor of Google [30, 31]. Potential solutions involve better education and better promotion all around, comments echoed by the EUAB. Librarians should tailor their instruction to each user group and consider limiting the number of resources demonstrated. Improving users' understanding of the resources should not be restricted to librarians. Publishers can create videos with product tours or share editorial purpose and highlights. Publishers can also be strategic, making sure the tools that are included are helpful to the user's experience.

**Integration** is a pain point that includes integrating information resources into clinicians' workflow. The integration pain point is closely linked to time because improved integration is a good way to improve efficiency. Information resources are not easily accessible in and around the patient encounter [32]. The advisory board mentioned issues in the number of steps that it took to get to resources that were already implemented and various barriers around accessing the full resource. The first step in mitigating this pain point could be in understanding where users begin their searches. If searches begin in the EHR, resource integration and access should be made easier, more visible, and more efficient [7, 17]. Improved and easier access via mobile devices, info or “easy” buttons, and integration of key resources like clinical practice guidelines were solutions that the EUAB offered. Other solutions included librarians advocating for the integration of the highest priority or most heavily used resources and publishers improving remote access for offsite and after hours use of information resources.

The final pain point is **financial limitations,** which touches on how financial issues can limit user access to resources [7]. The cost of resources influences access, and decisions regarding resource purchasing or subscriptions greatly impact the availability of specific resources for end users. The financial burden of acquiring information products or tools causes unequal access for clinical providers, a problem mentioned specifically by the EUAB members at nonacademic institutions. Another shared that there was high demand for free, reliable resources among nursing and allied health professionals. Potential solutions for this pain point are tricky because discussing finances is always a sticky subject, but more access can only improve patient care [4, 33]. Librarians in limited financial settings should search out and promote free resources that are accurate. Institutions and publishers need to come together for a more nuanced discussion of pricing for information tools and resources and develop creative solutions that benefit everyone.

Though the MLA InSight Initiative Summits 4 and 5 and conversations with the EUAB members took place before the COVID-19 pandemic, the working group has taken note of how industry partners have demonstrated that opening access to resources has made a significant impact on patient care and illustrates the importance of financial flexibility. More complex solutions involve analysis of users excluded in current pricing models and lobbying accrediting bodies for changes in issues around information resources.

## DISCUSSION

The working group created during MLA's InSight Initiative Summits 4 and 5 tackled pain points related to end user access to resources and information in a clinical environment. We found common ground during our discussions on supporting evidence-based medicine; on helping users find the most reliable, accurate information for clinical care; and on providing end users with assistance and training on how best to use information resources.

Early on in the process of identifying and defining pain points, the working group discussed potential “owners” for the solutions, such as the publisher, user, librarian, or institution. These owners would be responsible for providing effort toward solving the pain point. Depending on the pain point, some owners could have more responsibility for potential solutions. For example, the burden for resource scope would be on the librarian to educate and promote resources to users. However, we found that every owner could play at least a small role in providing solutions; therefore, we decided to focus on the impact the solutions could have on the end user.

The working group decided to mark each pain point with the level of impact (low, medium, high) that implementing a solution for the pain point might have on the end user. The group identified several issues such as search skills, privacy, and integration as having a high level of impact. But when the impact question for each issue was posed to the EUAB, they overwhelmingly identified time as the most acute pain point in which a solution would provide the highest level of impact. While complex access issues plague librarians and have thus far yielded the most debate and potential solutions, the end users we consulted did not feel the same way. They did not feel that potential solutions in this area would have a large impact on their work, while solutions around paywalls and integration could improve their workflow and potentially impact patient care.

## NEXT STEPS: CALL TO ACTION

We encourage librarians and industry members to consider how they might contribute to the mitigation of these proposed end user pain points. Our work was meant to draw attention to the issues that users face and indicate which issues they might consider most vexing. The framework and accompanying slides presented to the EUAB are available on MLANET. In addition, a library of references is available with useful links for each pain point area as well as some general references on information access.

The Insight Initiative Forum will be a virtual home for constructive dialogue between health sciences librarians and publishers about our shared interests in improving discovery of—and access to—information. The goals of the forum include problem solving, industry-wide communication, and sharing of feedback. It will be hosted by MLA and moderated by a team of volunteers.

The MLA InSight Initiative Learning content offers modules to educate librarians and publishers about critical aspects of user behavior. The modules are intended to be very brief, engaging, and digital, “ready-made” to be linked-to from websites and via social media. They can be used not only for teaching and instructional purposes, but also for orientations, promotional outreach, and during consultations. Early module topics under development include the risks of predatory publishing, quick connection to library resources, and use of interlibrary loan services.

The unique partnership between librarians and industry professionals at the InSight Initiative Summits have yielded both high-level discussions and practical tools to help people working in health sciences information better serve their users. We hope that summits 4 and 5, which focused on understanding end user behavior in a clinical environment, will spark additional discussions and solutions to improve the user experience.
